# Bone Marrow Stem Cell Treatment for Ischemic Heart Disease in Patients with No Option of Revascularization: A Systematic Review and Meta-Analysis

**DOI:** 10.1371/journal.pone.0064669

**Published:** 2013-06-19

**Authors:** Sheila A. Fisher, Carolyn Dorée, Susan J. Brunskill, Anthony Mathur, Enca Martin-Rendon

**Affiliations:** 1 Systematic Review Initiative, NHS Blood and Transplant, John Radcliffe Hospital, Oxford, United Kingdom; 2 Nuffield Division of Clinical Laboratory Sciences, Radcliffe Department of Medicine, University of Oxford, Oxford, United Kingdom; 3 Queen Mary University of London CV Biomedical Research Unit, Barts and The London School of Medicine and Dentistry, London, United Kingdom; 4 Stem Cell Research Laboratory, NHS Blood and Transplant, John Radcliffe Hospital, Oxford, United Kingdom; University of Nevada School of Medicine, United States of America

## Abstract

**Objective:**

To evaluate bone marrow stem cell treatment (BMSC) in patients with ischemic heart disease (IHD) and no option of revascularization.

**Background:**

Autologous BMSC therapy has emerged as a novel approach to treat patients with acute myocardial infarction or chronic ischemia and heart failure following percutaneous or surgical revascularization, respectively. However, the effect of the treatment has not been systematic evaluated in patients who are not eligible for revascularization.

**Methods:**

MEDLINE (1950–2012), EMBASE (1980–2012), CENTRAL (*The Cochrane Library* 2012, Issue 8) and ongoing trial databases were searched for relevant randomized controlled trials. Trials where participants were diagnosed with IHD, with no option for revascularization and who received any dose of stem cells by any delivery route were selected for inclusion. Study and participant characteristics, details of the intervention and comparator, and outcomes measured were recorded by two reviewers independently. Primary outcome measures were defined as mortality and measures of angina; secondary outcomes were heart failure, quality of life measures, exercise/performance and left ventricular ejection fraction (LVEF).

**Results:**

Nine trials were eligible for inclusion. BMSC treatment significantly reduced the risk of mortality (Relative Risk 0.33; 95% Confidence Interval 0.17 to 0.65; *P* = 0.001). Patients who received BMSC showed a significantly greater improvement in CCS angina class (Mean Difference −0.55; 95% Confidence Interval −1.00 to −0.10; *P* = 0.02) and significantly fewer angina episodes per week at the end of the trial (Mean Difference −5.21; 95% Confidence Interval −7.35 to −3.07; *P*<0.00001) than those who received no BMSC. In addition, the treatment significantly improved quality of life, exercise/performance and LVEF in these patients.

**Conclusions:**

BMSC treatment has significant clinical benefit as stand-alone treatment in patients with IHD and no other treatment option. These results require confirmation in large well-powered trials with long-term follow-up to fully evaluate the clinical efficacy of this treatment.

## Introduction

The incidence of ischemic heart disease (IHD) is increasing exponentially worldwide as a consequence of improved long-term survival following medical therapy and percutaneous or surgical revascularization procedures. Autologous bone marrow-derived stem cell (BMSC) therapy has emerged as a novel approach to treat patients with left ventricular dysfunction following acute myocardial infarction (AMI) despite successful revascularization by percutaneous coronary intervention (PCI) and patients with chronic ischemia and heart failure who have received surgical revascularization [1]–[4]. Globally, BMSC significantly improves left ventricular ejection fraction (LVEF) by 3–4% in patients who suffered from AMI [2], [5]. Phase I/II clinical trials have also been conducted administering BMSC as treatment for ischemic heart failure (HF). The treatment has proven to be safe and feasible and the treatment effect is promising [6]–[9]. However, some of the early studies comprise cohort studies that lack the appropriate control for the intervention [10], [11]. More recently, a number of randomized trials have treated patients with ischemic HF where revascularization procedures were administered concomitantly [6]–[9]. Considering that revascularization procedures have improved the management and long-term outcome of IHD greatly, it becomes more difficult to assess the benefits of BMSC treatment when administered as a co-intervention. Recently, several randomized controlled trials (RCTs) have tested BMSC as treatment for those patients receiving maximal medical therapy, with symptoms of intractable angina or HF and where patients were not eligible for revascularization [12]–[20]. We consider that the evaluation of BMSC treatment as a stand-alone therapy is critical and may be beneficial for those patients who have exhausted all conventional therapies and where revascularization is no longer an option due to the lack of suitable conduit vessels or the diffuse nature of the disease. Here we present a systematic review and meta-analysis of autologous BMSC treatment in this cohort of patients. In this study, mononuclear cells harvested by density gradient centrifugation or leukapheresis, and/or enriched in hematopoietic stem cells (e.g. CD34-positive or Aldehyde Dehydrogenase (ALDH)-positive cells) by magnetic cell separation or cell sorting are referred to as BMSC.

## Methods

### Eligibility

Inclusion criteria were as follows: (i) randomized controlled trials (RCTs), (ii) participants with no option for percutaneous or surgical revascularization, diagnosed with IHD, with symptoms of angina or HF according to the Canadian Cardiology Society (CCS class II–IV) and New York Heart Association (NYHA class II–IV), and receiving maximal medical treatment, (iii) any dose of BMSC, (iv) any delivery route, and (v) any other co-intervention provided it was administered equally to all arms in the trial. BMSC were defined as mononuclear cells that were harvested by density gradient centrifugation or leukapheresis, and in some cases further enriched in hematopoietic stem cells (CD34-positive or ALDH-positive cells) by magnetic cell separation or cell sorting prior to administering them to participants. Exclusion criteria: trials involving participants with IHD who were eligible for revascularization by percutaneous or surgical procedures. RCTs included administered cells harvested from the bone marrow or from peripheral blood after bone marrow mobilization, referred to here as bone marrow-derived stem cells.

### Search Strategy

MEDLINE (1950–2012), EMBASE (1974–2012), CENTRAL (*The Cochrane Library* 2012, Issue 8), CINAHL (1982–2012), PUBMED (epublications only), LILACS, KOREAMED, INDMED, PAKMEDINET, and the Transfusion Evidence Library were searched through to 21st August 2012 for RCTs that follow the inclusion criteria detailed above. Ongoing trial registers (ClinicalTrials.gov, the ISRCTN Register, the World Health Organisation International Clinical Trials Platform Registry, UMIN-CTR Japanese Clinical Registry and the Hong Kong Clinical Trials Registry) were also searched. Searches were combined with adaptations of the Cochrane highly sensitive RCT search filter in MEDLINE, EMBASE and CINAHL. Proceedings from the American Heart Association (2005–2011) and European Society of Cardiology (2005–2011) conferences and the reference lists of identified studies and relevant review articles were handsearched for additional studies. No restriction by language, year of publication or publication status was applied. Detailed search strategies are available from the authors upon request.

### Data Extraction and Quality Assessment

Eligibility screening, data extraction and assessment of methodological quality were undertaken by two independent reviewers. Discrepancies were resolved by consensus. Data extracted from included studies were as follows: (i) characteristics of the patient population and the study, (ii) type of intervention and comparator, and (iii) outcomes measured. Primary outcome measures were defined as mortality and measures of angina (CCS class and frequency of angina episodes). Secondary outcomes included NYHA class, quality of life (QoL) measures, exercise/physical performance, LVEF and myocardial perfusion.

Assessment of the quality of studies was made according to The Cochrane Collaboration's tool for assessing risk of bias in randomized trials which is based on the generation of random sequence, concealment of treatment allocation, blinding of participants, clinicians and outcome assessors and loss to follow-up [21].

### Statistical Analysis

RevMan 5.1 [22] was used to analyze outcome data. Dichotomous outcomes are presented as Relative Risk (RR) with 95% Confidence Intervals (CI). For continuous outcomes, the mean change from baseline over the study follow-up period was the preferred measure of outcome; the mean value at endpoint was used where insufficient data were available to calculate the mean change from baseline. Where standard deviations were not explicitly reported, these were estimated where possible from reported *P* values or CIs. Continuous outcomes are presented as mean difference (MD) between treatment groups with a 95% CI. For QoL and performance measures, the standardized MD (SMD) was used in order to allow analyses of outcomes measured on different scales. Meta-analyses were performed using fixed effect models, except when a high degree of heterogeneity was observed, where random effects models were used. The I^2^ statistic [23], [24] was used to evaluate statistical heterogeneity, where an I^2^ statistic >75% denotes high heterogeneity [24]. P<0.05 was considered statistically significant; two-sided significance values are reported throughout.

## Results

### Description of the included studies

**Figure 1:**
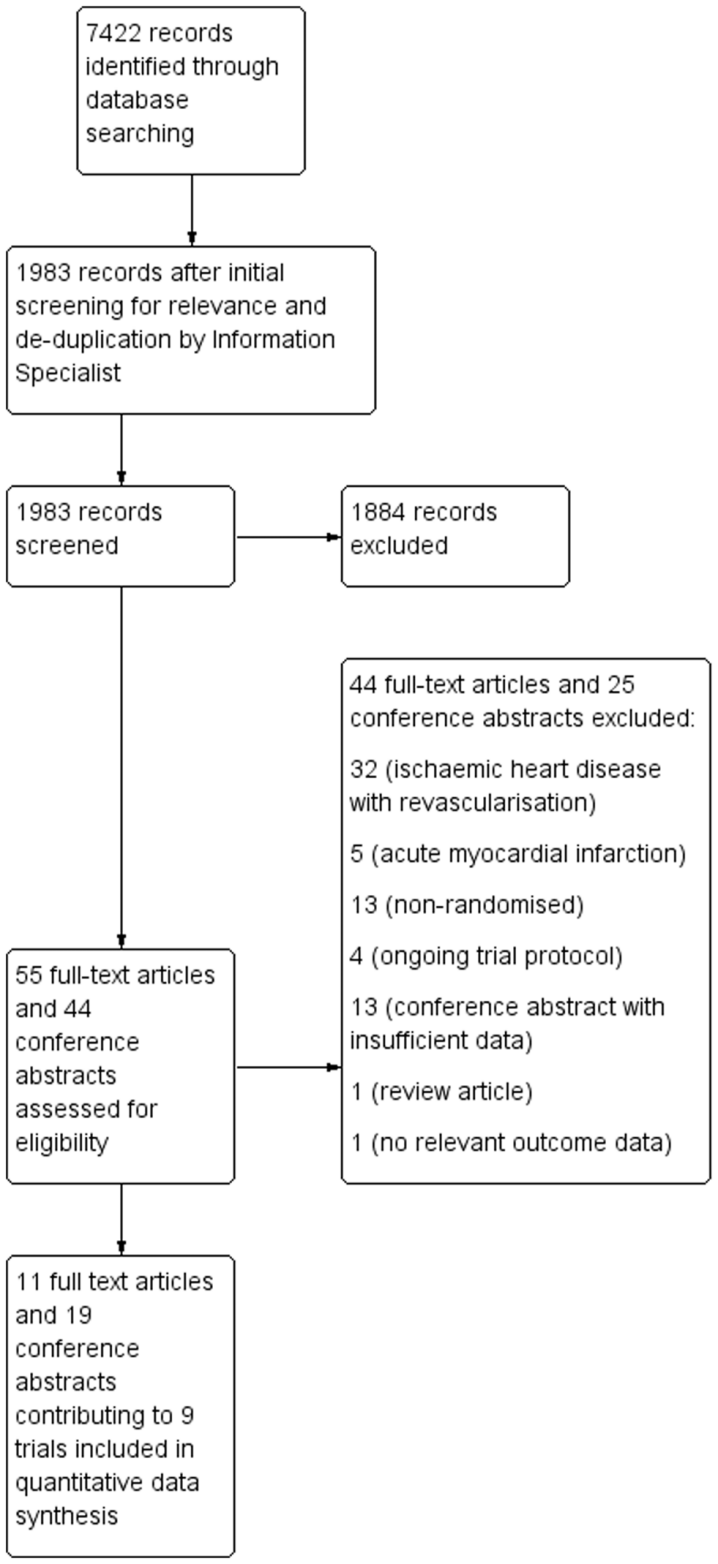
PRISMA flow diagram of study selection.

A total of 7422 citations were identified initially (Figure 1) using the search strategies detailed in Methods S1; these were reduced to 1983 citations after removal of duplicates and preliminary screening for relevance by the Information Specialist. Screening of these 1983 citations by two reviewers, independently and in duplicate, eliminated a further 1884 records. The remaining 99 citations (55 full text articles and 44 conference abstracts) were assessed for eligibility. From these, 69 citations were excluded as they did not fully meet the inclusion criteria (Figure 1). The remaining 11 full text articles and 19 conference abstracts contributed to nine independent trials [12]–[26] included in this systematic review. The characteristics of the included studies are shown in Table S1.

In one study [13], patients received one of three treatment arms: either a placebo or one of two doses of mobilized autologous CD34+ cells (1×10^5^ or 5×10^5^ cells/kg); these are denoted low dose (LD) and high dose (HD). To avoid double counting of the control group which can result in correlated results between the two treatment arms, this trial was analysed by two different methodological approaches. Firstly, data from the two BMSC treatment groups were pooled into a single trial arm and compared with the control group; secondly, the control group was divided into two groups of equal size and effect, allowing for separate analysis of each BMSC treatment group. Results for both methods were similar for all analyses (data not shown) and therefore, results for this study are presented for each BMSC treatment group separately with an equally divided control group providing a comparator arm for both treatment groups.

A second study [12] randomized patients to one of four treatment arms: either a placebo or one of four doses of mobilized autologous CD34+ cells (5×10^4^, 1×10^5^ or 5×10^5^ cells/kg). However, no dose-response effect was found in this trial and results were therefore reported for the combined treatment groups.

The included trials compared BMSC treatment to control in a total of 659 patients (363 BMSC and 296 controls) (see Table S1). Four trials treated patients with ischemic HF [14]–[17] whilst five trials treated patients with intractable angina [12], [13], [18]–[20]. Study sample sizes ranged from 10 to 56 for BMSC and from 6 to 56 for controls.

Five of the nine included studies harvested BMSC directly from the bone marrow by aspiration and enriched the cell fraction with mononuclear cells by density gradient centrifugation [14], [15], [17]–[19]. Two trials [12], [13] treated patients in the control and treatment groups with granulocyte colony stimulating factor (G-CSF) prior to isolating BMSC from peripheral blood by leukapheresis and enriching the BMSC population in CD34-positive cells by magnetic separation. In a third trial [20], patients were treated with G-CSF and CD34-positive cells isolated from bone marrow aspirates. One trial [16] enriched the bone marrow mononuclear cell fraction in ALHD-positive cells by cell sorting prior to administration. All trials maintained the patients under maximal standard medication throughout the trials.

All trials presented outcome measures within six months follow-up and two [13], [17] conducted longer follow-up of patients, up to 12 months.

### Methodological quality assessment of included studies

Overall, the methodological quality of the included trials was good. Treatment was randomized in all studies; adequate methods of randomization sequence generation were employed in all but two trials [12], [20] in which the method of randomization was not reported, and concealment of treatment allocation was unclear in two trials [12], [17]. Methods of randomization included computer generated randomization sequences or codes generated from randomization tables which were distributed using numbered sealed envelopes. In one study [13], treatment was assigned by the cell-processing laboratory using a telephone call-in and interactive voice-response system. In all but one study [17], controls received a placebo, or in one case, a simulated mock injection procedure [14]. Outcome assessors were blinded to treatment allocation in all trials. At least 89% (range 89% to 100%) of randomized participants were included in the analysis of the study primary outcome in all but one study [17]. In this study of patients with end-stage chronic HF, a high rate of mortality (25% of all participants) was observed during the follow-up period. Four of the included studies [13], [15], [18], [19] reported a power calculation to determine the sample size required to show a significant effect of the primary outcome.

### Primary outcomes

#### (i) Mortality

**Figure 2:**
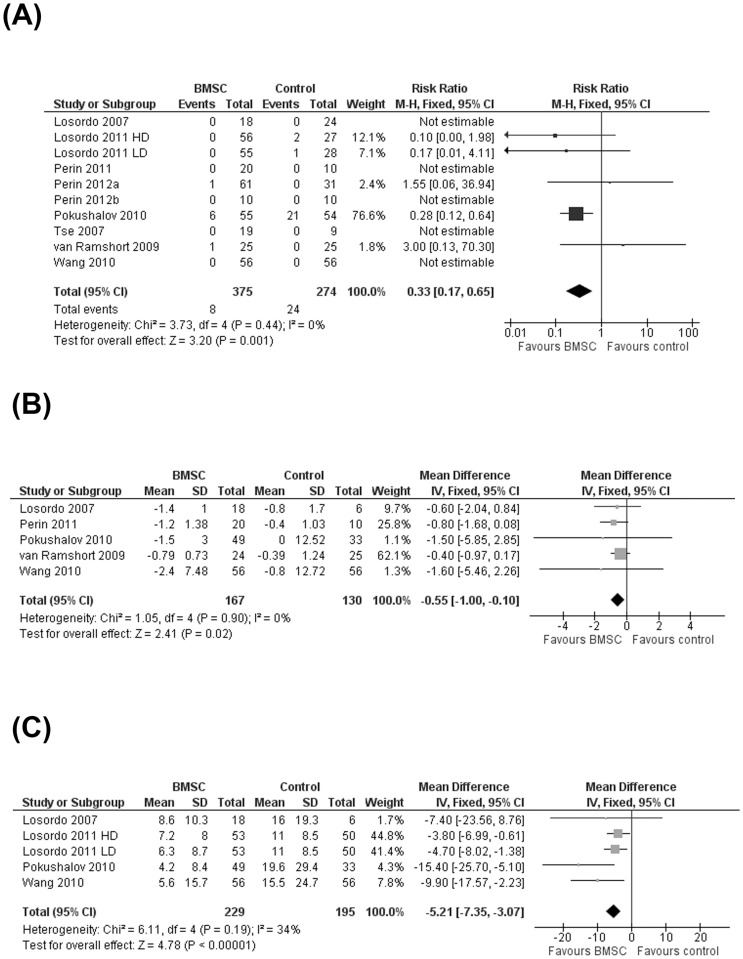
Effect of bone marrow stem cell on primary outcomes. (A) Risk ratio of mortality, (B) mean change in angina class (CCS class) from baseline to end of study and (C) mean change in angina frequency (number of episodes per week) at the end of study.

All included trials reported mortality due to any cause. Five trials [12], [14], [16], [18], [20] reported no incidence of mortality throughout the follow-up period. One trial [17] observed a high incidence of mortality (BMSC: 10.9%; controls: 38.9%) during the trial; this was likely due to the severe end-stage HF diagnosis of the patients in this study compared with the other included studies. In the remaining three trials, a total of three deaths were observed in untreated patients [13] compared with two deaths in the BMSC group [15], [19]. Of the two deaths in patients who received BMSC treatment, one was due to acute HF [19], the other was deemed unlikely to be associated with cell therapy [15]. The reasons for mortality in the three control group participants were not reported [13]. Meta-analysis of all trials which reported incidence of mortality showed a significantly reduced risk of mortality in patients who received BMSC compared with controls (RR 0.33; 95% CI 0.17 to 0.65; *P* = 0.001) (Figure 2A).

Six trials [12], [14], [16], [18]–[20] reported mortality due to reinfarction as an outcome; none of these reported any incidence of mortality due to reinfarction during the trial follow-up period. However, in one trial [18], one death at 31 months due to reinfarction was reported in an individual who received placebo. Eight trials [12]–[16], [18]–[20] reported morbidity of myocardial infarction as an outcome; four of these trials [12], [14], [19], [20] reported no incidence of myocardial infarction throughout the trial. Meta-analysis of the four trials which reported myocardial infarction in at least one of the treatment arms showed a reduced risk of myocardial infarction associated with BMSC although this failed to reach statistical significance (RR 0.55; 95% CI 0.24 to 1.27; *P* = 0.16).

#### (ii) Measures of angina

Clinical angina status, according to the CCS angina class, was reported as an outcome in all studies. However, one trial [13] only reported the percentage of patients in each treatment group with a change (improvement or worsening) in CCS class. In particular, this study reported a ≥2-class improvement in CCS class over the length of the trial in 23.1% of LD and 25.0% HD treated patients compared with only 15.2% of controls. Another trial [15] reported only that there were “no significant differences in the change in CCS class”. Two further studies [16], [18] only reported CCS class at the end of the trial and insufficient data were reported to enable calculation of standard deviations of the mean change from baseline values; neither of these trials reported a significant difference in CCS class between treatment groups at the end of the trial. Meta-analysis of the five remaining trials [12], [14], [17], [19], [20] showed a significant difference in mean change from baseline between groups in favor of BMSC (MD −0.55; 95% CI −1.00 to −0.10; p = 0.02) (Figure 2B).

Angina frequency (number of episodes per week) was measured in four trials [12], [13], [17], [20], although only two trials [12], [20] reported mean change in angina frequency from baseline and therefore, angina frequency was compared between treatment groups at the end of the trial. Meta-analysis of the number of angina episodes per week revealed a significant difference between treatment groups in favor of BMSC (MD −5.21; 95% CI −7.35 to −3.07; p<0.00001) (Figure 2C).

### Secondary outcomes

#### (i) Functional status for heart failure (NYHA class)

Functional data on HF, according to NYHA classification, was measured in four studies [14], [16]–[18] Mean change in NYHA class from baseline to the end of the trial was not reported in two studies and therefore, NYHA class was compared between treatment groups at the end of the trial. NYHA class was significantly lower in patients treated with BMSC than in controls in all but one study [16]. High heterogeneity was observed across studies (I^2^ = 98%; 95% CI 96.7% to 98.8%). Pooled evidence across studies using a random effects model showed a lower NYHA class at the end of the trial in patients who received BMSC compared with controls, although this difference did not reach statistical significance (MD −0.56; 95% CI −1.29 to 0.17; *P* = 0.13).

#### (ii) QoL

**Figure 3:**
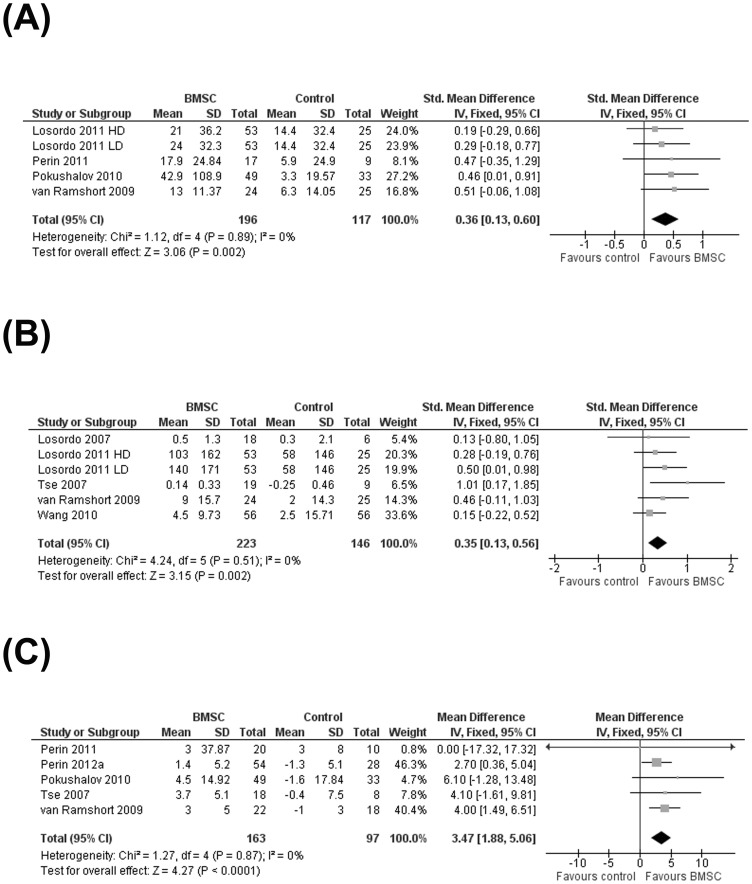
Effect of bone marrow stem cell on the secondary outcomes. (A) Standardized mean change in Quality of Life (QoL), (B) exercise/physical performance, and (C) mean difference in left ventricular ejection fraction.

Patient-reported quality-of-life measures were reported in four studies. These included the Seattle Angina Questionnaire [13], [19], Minnesota Living with Heart Failure (MLHF) Questionnaire [14], [17] and SF-36 Health Survey [14]. All four studies observed an improvement in QoL in patients who received BMSC compared with controls although this improvement was only statistically significant in one trial [17]. In order to assess QoL measures across all studies simultaneously, Seattle Angina Questionnaire and MLHF Questionnaire data were combined in a meta-analysis (as described in the Methods section). Pooled evidence across studies showed a significant improvement in QoL (SMD 0.36; 95% CI 0.13 to 0.60; *P* = 0.002) (Figure 3A).

#### (iii) Performance and exercise

Performance and exercise capacity measures were reported in six studies. Measures included a standard Bruce protocol treadmill exercise tolerance test [12], [20], a modified Bruce protocol treadmill exercise tolerance test [13], [18], a six minute walking test [17] and a symptom-limited bicycle exercise test [19]. One study [17] did not report sufficient data to calculate mean change from baseline data, although this study did report a significant increase from baseline in the distance walked at the end of the trial in patients who received BMSC but not in controls. Pooled data from the remaining five studies showed a significant improvement in exercise performance in patients who received BMSC compared with controls (SMD 0.35; 95% CI 0.13 to 0.56; *P* = 0.002) (Figure 3B).

#### (iv) Heart contractility: left ventricular ejection fraction

Six studies [14]–[19] reported LVEF (%) although one study [16] did not provide sufficient data to calculate the standard deviation of the mean change from baseline. Of the the remaining five studies, only two [15], [19] reported a significant difference in mean change in LVEF from baseline in patients who received BMSC compared with controls. However, when data from all five studies were pooled, the combined evidence across studies showed a significant difference in mean change from baseline between treatment groups, in favor of BMSC (MD 3.47; 95% CI 1.88 to 5.06; *P* = 0.00002) (Figure 3C).

#### (v) Myocardial perfusion

Myocardial perfusion was measured by single-photon emission computed tomography (SPECT) imaging and reported in all included trials. However, there was low consistency in the methods and extent of reporting (automated versus visual interpretation, stress/rest/total defect size, summed stress/rest/difference scores) and therefore, no formal statistical comparison of results could be made. Nevertheless, seven of the nine trials reported a greater improvement in at least one measure of myocardial perfusion in patients who received BMSC compared with controls.

## Discussion

We present here the results of a systematic review and meta-analysis of RCTs where autologous BMSC treatment is administered to patients with IHD who are not eligible for revascularization. To our knowledge, this is the first systematic review and meta-analysis that evaluates BMSC treatment as stand-alone therapy.

In all trials except one, the cells were delivered into viable myocardium using electromechanical mapping and the effect of intramyocardial injection was controlled by a mock injection in the placebo/control arm of the trial. Our previous work suggests that this delivery method is more effective that intracoronary infusion (5). These trials used mostly bone marrow mononuclear cells with the exception of three in which CD34-positive cells were enriched and one in which ALDH-positive cells were sorted, all from the mononuclear cell fraction, but the dose administered in each trial varied. Altogether, our data suggest that BMSC treatment is safe and significantly reduces mortality and angina in patients with no other treatment option. These results are extremely encouraging and potentially very important in this cohort of patients. Other trials in which bone marrow-derived mesenchymal stem cells are modified during culture to induce a cardiogenic phenotype are underway [27] (abstr). In the future, it will be interesting to evaluate whether different cell populations and different cell processing methods may yield comparable treatment effects.

All nine included trials reported mortality as an outcome, although only five trials reported any incidence of mortality in either trial arm and only these trials contributed to the risk ratio (RR) estimate (Figure 1A). The dramatic reduction in mortality may be due to the cohort of patients included (angina and/or HF according to NYHA and CCS class II–IV). In particular, one study [17] recruited patients with HF at a more advanced stage (NYHA class III–IV). Although the results of this meta-analysis suggest a significant reduction of mortality, they have to be considered with caution and confirmed in larger clinical trials. Reduction of mortality has been demonstrated in very few trials and never in a meta-analysis of AMI studies [2].

A significant reduction in angina symptoms was demonstrated using two measures of angina: CCS class and frequency of episodes per week. Meta-analysis of an aggregate measure of angina was not performed since the mean change from baseline was not reported in all trials. Additionally, the risk estimate obtained using the SMD, a method which can be used to analyse aggregate measures, has limited interpretation value. However, the significant mean difference between treatment and control arms for both measures provides compelling evidence that BMSC reduces symptoms of angina.

The included trials fall into two categories: those treating patients with ischemic HF and those treating refractory angina patients, and their primary outcome differs depending on the objective of the trial. Interestingly, and despite this clinical heterogeneity, the present study shows that statistical heterogeneity among included trials is low for most of the outcomes measured, suggesting that the treatment has very similar effect on all the trials. This conflicts with the high heterogeneity observed in our previous meta-analysis where only AMI trials were included [1], [2] (e.g. LVEF, I^2^ = 73% (95% CI: 63.7% to 79.4%)) and in a recent systematic review that identified 50 trials of BMSC treatment in IHD [3] and combined RCTs with cohort studies as well as AMI and chronic IHD and HF (e.g. LVEF, I^2^ = 80% (95% CI: 72.9% to 85.2%)). Whilst the current study has lower statistical power to detect heterogeneity due to the lower number of included studies, the upper confidence limit of I^2^ for LVEF and other outcomes suggests that at most, moderate heterogeneity exists between these studies [24]. Conversely, the large number of studies included in the previous meta-analyses provided high statistical power for detecting heterogeneity [1]–[3]. These discrepancies may be explained by, but not limited to, (i) revascularization procedures being a source of variability during treatment and/or (ii) the administration of cells into viable myocardium leading to a more efficient and more efficacious delivery method.

The present study shows that BMSC treatment also significantly improves QoL and performance status compared to controls. Studies with participants with symptoms of angina and/or HF according to NYHA and CCS class II–IV were eligible for inclusion. The high heterogeneity observed for NYHA class may be due to different baseline values in some studies.

For outcomes measured on different scales such as QoL and exercise performance measures described here, the standardized mean difference (SMD) provides a useful method of standardizing measurements into a uniform scale so that they can be pooled in a meta-analysis. As noted above, results from a meta-analysis of measurements on different scales using the SMD can be difficult to interpret as the effect of the intervention is expressed in standardized units rather than the original units of measurements. A study which evaluated the correlation (Pearson's correlation coefficient, r) between MLHF questionnaire and Seattle Angina Questionnaire showed a highly significant correlation between these measures in patients with both angina (r = 0.826) and HF (r = 0.821) [28]. Nevertheless, the interpretation of results from such an analysis using standardized measures should be treated with caution.

The results of this study also suggest that BMSC treatment significantly improves global LVEF by 3–4%. As a stand-alone therapy, BMSC seems to have a beneficial effect on global heart contractility as previously observed [2], [4], [29].

The present systematic review and meta-analysis has a number of advantages over previous ones. Firstly, it evaluates the effect of BMSC treatment in a cohort of patients who suffer from IHD and are not eligible for percutaneous or surgical revascularization. Secondly, it assesses the effect of treatment on clinical outcomes such as HF and angina symptoms, QoL and physical exercise/performance, that have not been fully evaluated previously. These clinical outcomes are extremely important in the management of the disease in this cohort as the patients have no other treatment option.

The present study has two main limitations. First, the small number of trials included and the size of the trials require that conclusions from this systematic review and meta-analysis are considered cautiously and may need to be substantiated with larger clinical trials. Moreover, this is also a restriction in conducting further sensitivity analyses, investigating potential sources of heterogeneity and evaluating publication bias. Second, the length of follow-up in the included studies is relatively short (6 and 12 months). The field would benefit from long-term follow-up to confirm the efficacy of this treatment.

In conclusion, we have demonstrated a significant beneficial effect of BMSC as a stand-alone treatment for patients with IHD without the option of revascularization and where cells are injected into viable myocardium. Larger well-powered trials with long-term follow-up will be needed to fully evaluate the efficacy of BMSC and the optimal delivery method in this cohort of patients.

## Supporting Information

Methods S1 **Search strategies.**
(DOC)

Table S1 **Characteristics of included studies.**
(DOCX)
