# Supplementary material for: Bone Marrow Stem Cell Treatment for Ischemic Heart Disease in Patients with No Option of Revascularization: A Systematic Review and Meta-Analysis
Source: PLoS One. 2013 Jun 19;8(6):e64669. doi: 10.1371/journal.pone.0064669 (PMC3686792; doi:10.1371/journal.pone.0064669)
Supplement: Methods S1 — Search strategies. (DOC) [file pone.0064669.s001.doc]

**Methods S1: SEARCH STRATEGIES**

**THE COCHRANE LIBRARY**

#1 STEM CELL TRANSPLANTATION explode all trees (MeSH)

#2 HEMATOPOIETIC STEM CELL MOBILIZATION single term (MeSH)

#3 STEM CELLS explode all trees (MeSH)

#4 CELL TRANSPLANTATION single term (MeSH)

#5 haematopoietic or hematopoietic or haematopoetic or hematopoetic or hemopoietic or haemopoietic or marrow NEAR cell* or stem cell* or progenitor cell* or precursor cell* or cell* therapy

#6 ((myoblast* or cell*) NEAR (transplant* or graft* or implant*))

#7 #1 or #2 or #3 or #4 or #5 or #6

#8 MYOCARDIAL ISCHEMIA explode all trees (MeSH)

#9 HEART FAILURE explode all trees (MeSH)

#10 HEART DISEASES single term (MeSH)

#11 (myocardial or myocardium or subendocardial or transmural or cardiac or cardial or coronary or heart) NEAR (infarct* or postinfarct* or hypoxi* or anoxi* or failure* or decompensation or insufficien*)

#12 heart disease* or coronary disease* or IHD or CIHD

#13 chronic myocardial dysfunction or angina or stenocardia

#14 (ischemi* or ischaemi*) NEAR (myocardium or myocardial or heart or coronary or cardiac or cardial or subendocardial or cardiomyopath*)

#15 (artery occlusion* or artery disease* or arterioscleros* or atheroscleros*) NEAR coronary

#16 (heart or cardiac or cardial or myocardium or myocardial) NEAR (repair* or reparation or improve* or regenerat*)

#17 #8 or #9 or #10 or #11 or #12 or #13 or #14 or #15 or #16

#18 #7 AND #17

#19 (cellular NEXT cardiomyoplast*) or (cardiomyocyte* NEAR transplant*) or (intramyocardial* NEAR (transplant* or stem or bone marrow)) or (transendocardial* NEAR stem NEXT cell*) or (intracoronary NEAR progenitor NEXT cell*) or (transcoronary NEAR transplant*)

#20 #18 or #19

**MEDLINE (Ovid)**

1. CELL TRANSPLANTATION/

2. exp STEM CELL TRANSPLANTATION/

3. BONE MARROW TRANSPLANTATION//

4. exp STEM CELLS/

5. (haematopoietic or hematopoietic or haematopoetic or hematopoetic or hemopoietic or haemopoietic or (marrow adj2 cell*) or stem cell* or progenitor cell* or precursor cell* or cell* therapy or bone marrow).ti,ab.

6. ((mesenchymal or stromal) AND marrow).ti,ab.

7. (cell*) adj3 (transplant* or graft* or implant*)).ti,ab

8. cell transplantation.jn. or cell stem cell.jn. or stem cell reviews.jn. or bone marrow transplantation.jn.

9. or/1-8

10. exp MYOCARDIAL ISCHEMIA/

11. exp HEART FAILURE/

12. HEART DISEASES/

13. ((myocardial or myocardium or subendocardial or transmural or cardiac or cardial or coronary or heart) adj2 (infarct* or postinfarct* or hypoxi* or anoxi* or failure* or decompensation or insufficien*)).ti,ab.

14. (heart disease* or coronary disease* or IHD or CIHD).ti,ab.

15. (chronic myocardial dysfunction or angina or stenocardia).ti,ab.

16. ((ischemi* or ischaemi*) adj2 (myocardium or myocardial or heart or coronary or cardiac or cardial or subendocardial or cardiomyopath*)).ti,ab.

17. ((end stage or endstage) adj cardiomyopath*).ti,ab.

18. ((artery occlusion* or artery disease* or arterioscleros* or atheroscleros*) adj2 coronary).ti,ab.

19. ((heart or cardiac or cardial or myocardium or myocardial) adj3 (repair* or reparation or improve* or regenerat*)).ti,ab.

20. or/10-19

21. 9 and 20

22. ((cellular adj cardiomyoplast*) or (cardiomyocyte* adj5 transplant*) or (intramyocardial* adj6 (transplant* or stem or bone marrow)) or (transendocardial* adj5 stem adj cell*) or (intracoronary adj5 progenitor adj cell*) or (transcoronary adj3 transplant*)).mp.

23. 21 or 22

**EMBASE (Ovid)**

1. exp CELL THERAPY/

2. exp STEM CELL/

3. BONE MARROW CELL/

4. ((mesenchymal or stromal) AND marrow).ti,ab.

5. (haematopoietic or hematopoietic or haematopoetic or hematopoetic or hemopoietic or haemopoietic or marrow adj2 cell* or stem cell* or progenitor cell* or precursor cell* or cell* therapy or bone marrow).ti,ab.

6. (cell* adj3 (transplant* or graft* or implant*)).ti,ab.

7. cell transplantation.jn. or cell stem cell.jn. or stem cell reviews.jn.

8. or/1-7

9. exp ISCHEMIC HEART DISEASE/

10. exp HEART FAILURE/

11. exp MYOCARDIAL DISEASE/

12. ((myocardial or myocardium or subendocardial or transmural or cardiac or cardial or coronary or heart) adj2 (infarct* or postinfarct* or hypoxi* or anoxi* or failure* or decompensation or insufficien*).ti,ab.

13. (heart disease* or coronary disease* or IHD or CIHD).ti,ab.

14. (chronic myocardial dysfunction or angina or stenocardia).ti,ab.

15. ((ischemi* or ischaemi*) adj2 (myocardium or myocardial or heart or coronary or cardiac or cardial or subendocardial or cardiomyopath*)).ti,ab.

16. ((artery occlusion* or artery disease* or arterioscleros* or atheroscleros*) adj2 coronary).ti,ab.

17. ((end stage or endstage) adj cardiomyopath*).ti,ab.

18. ((heart or cardiac or cardial or myocardium or myocardial) adj3 (repair* or reparation or improve* or regenerat*)).ti,ab.

19. or/9-18

20. 8 AND 19

21. ((cellular adj cardiomyoplast*) or (cardiomyocyte* adj5 transplant*) or (intramyocardial adj6 (transplant* or stem or bone marrow)) or (transendocardial adj5 stem adj cell*) or (intracoronary adj5 progenitor adj cell*) or (transcoronary adj3 transplant*)).mp.

22. 20 or 21

**CINAHL (NHS Evidence)**

1. exp CELL TRANSPLANTATION/

2. exp STEM CELLS/

3. (haematopoietic OR hematopoietic OR haematopoetic OR hematopoetic OR hemopoietic OR haemopoietic OR (marrow adj2 cell*) OR "stem cell*" OR "progenitor cell*" OR "precursor cell*" OR "cell* therapy" OR "bone marrow").ti,ab

4. ((mesenchymal or stromal) AND marrow).ti,ab

5. ((cell* adj3 transplant*) OR (cell* adj3 graft*) OR (cell* adj3 implant*)).ti,ab

6. 1 or 2 or 3 or 4 or 5

7. exp MYOCARDIAL ISCHEMIA/

8. exp HEART FAILURE, CONGESTIVE/

9. HEART DISEASES/

10. ((myocardial or myocardium or subendocardial or transmural or cardiac or cardial or coronary or heart) AND (infarct* or postinfarct* or hypoxi* or anoxi* or failure* or decompensation or insufficien*).ti,ab

11. ("heart disease*" or "coronary disease*" or IHD or CIHD).ti,ab

12. ("chronic myocardial dysfunction" OR angina OR stenocardia).ti,ab

13. ((ischemi* or ischaemi*) AND (myocardium or myocardial or heart or coronary or cardiac or cardial or subendocardial or cardiomyopath*)).ti,ab

14. ((chronic or artery occlusion* or artery disease* or arterioscleros* or atheroscleros*) AND coronary).ti,ab

15. ((heart or cardiac or cardial or myocardium or myocardial) AND (repair* or reparation or improve* or regenerat*)).ti,ab

16. 7 or 8 or 9 or 10 or 11 or 12 or 13 or 14 or 15

17. 6 AND 16

18. ((cellular adj2 cardiomyoplast*) or (cardiomyocyte* adj5 transplant*) or (intramyocardial* adj6 transplant*) or (intramyocardial* adj6 stem) or (intramyocardial* adj6 bone marrow)) or (transendocardial* adj5 “stem cell*”) or (intracoronary adj5 “progenitor cell*”) or (transcoronary adj3 transplant*))

19. 17 or 18

**TRANSFUSION EVIDENCE LIBRARY** (www.transfusionevidencelibrary.com)

("marrow cell*" OR "stem cell*" OR "progenitor cell*" OR "precursor cell*") AND (infarct* OR coronar* OR myocard* OR heart OR cardiac* OR cardiomyo* OR intramyocardial* OR ischemi* OR ischaemi* OR angina)

**PUBMED**

(stem[TI] OR marrow[TI] OR progenitor[TI] OR precursor[TI] OR cell[TI] OR cells[TI]) AND (infarct*[TI] OR coronar*[TI] OR heart*[TI] OR myocard*[TI] OR cardial[TI] OR cardiac[TI] OR transmural*[TI] OR ischemia[TI] OR ischemic[TI] OR subendocardial[TI] OR cardiomyopath*[TI] OR angina[TI]) AND (random* OR blind* OR control* OR placebo OR trial) AND (publisher[sb] NOT pubstatusnihms)

**LILACS**

("marrow cell$" OR "stem cell$" OR "progenitor cell$" OR "precursor cell$") AND (infarct$ OR coronar$ OR myocard$ OR heart OR cardiac$ OR cardiomyo$ OR intramyocardial$ OR ischemi$ OR ischaemi$ OR angina)

**KOREAMED**

(stem or marrow or progenitor or precursor or cell*) AND randomi*

**INDMED**

(marrow cell$ OR stem cell$ OR progenitor cell$ OR precursor cell$) AND (infarct$ OR coronar$ OR myocard$ OR heart OR cardiac$ OR cardiomyo$ OR intramyocardial$ OR ischemi$ OR ischaemi$)

**CURRENT CONTROLLED TRIALS (ISRCTN REGISTER)**

("stem cells" or "stem cell" or marrow or "progenitor cells" or "precursor cells") and (infarction or infarct or coronary or myocardial or heart or myocardium or cardial or transmural or ischemia or ischemic or subendocardial or cardiomyopathy OR angina)

**CLINICALTRIALS.GOV**

Study Type: Intervention Studies

Conditions: infarction OR infarct OR coronary OR myocardial OR heart OR myocardium OR cardial OR cardiac OR transmural OR ischemia OR ischemic OR subendocardial OR cardiomyopathy OR angina

Search Terms**:** marrow OR stem OR cell or cells OR myoblast OR myocell

**ICTRP**
Title: marrow OR stem OR hematopoietic OR precursor OR progenitor OR myoblast OR myocell OR mononuclear OR cells or cell

Condition: infarction OR infarct OR coronary OR myocardial OR heart OR myocardium OR cardial OR cardiac OR transmural OR ischemia OR ischemic OR subendocardial OR cardiomyopathy OR angina

Recruitment Status: ALL
