# Supplementary material for: Bone Marrow Stem Cell Treatment for Ischemic Heart Disease in Patients with No Option of Revascularization: A Systematic Review and Meta-Analysis
Source: PLoS One. 2013 Jun 19;8(6):e64669. doi: 10.1371/journal.pone.0064669 (PMC3686792; doi:10.1371/journal.pone.0064669)
Supplement: Table S1 — Characteristics of included studies. (DOCX) [file pone.0064669.s002.docx]

**Table S1: Characteristics of the included studies.**

| **Trial** | **Co- intervention** | **Type of stem cell** | **Mean dose (SD) of cells** | **Method of stem cell isolation and route of delivery** | **Comparator arm** | **Number of patients assessed for primary outcome** | | **Baseline Functional Class** | | **Baseline LVEF (%)**  **Mean (SD)** | | **Method(s) used to measure LVEF** | **Duration of trial** |
| --- | --- | --- | --- | --- | --- | --- | --- | --- | --- | --- | --- | --- | --- |
|  |  |  |  |  |  | **SC arm** | **Control arm** | **SC arm** | **Control arm** | **SC arm** | **Control arm** |  |  |
| Losordo 2007 | Medical therapy  +  G-CSF | CD34+ cells | 2.2 (2.5)x 10^5^ CD34+ cells/kg | Leukapheresis and selection of CD34 by MACS,  IM (EMM) | Injection of saline with 5% autologous plasma | 18 at 3 months  18 at 6 months | 6 at 3 months  6 at 6 months | NYHA: NR  CCS:  NR | NYHA:  NR  CCS:  NR | NR | NR | Not applicable | 6 m |
| Losordo LD 2011 | Medical therapy  +  G-CSF | CD34+ cells | 1 x 10^6^ CD34+ cells/kg | Leukapheresis and selection of CD34 by MACS,  IM (EMM) | Injection of saline with 5% autologous plasma | 54 at 6 months; 53 at 12 months | 53 at 6 months; 50 at 12 months† | NYHA: NR  CCS:  NR | NYHA:  NR  CCS:  NR | 58.9 (14.2) | 59.8 (14.5) | Echo or SPECT | 12 m |
| Losordo HD 2011 | Medical therapy  +  G-CSF | CD34+ cells | 5 x 10^6^ CD34+ cells/kg | leukapheresis and selection of CD34 by MACS,  IM (EMM) | Injection of saline with 5% autologous plasma | 55 at 6 months; 53 at 12 months | 53 at 6 months; 50 at 12 months† | NYHA: NR  CCS:  NR | NYHA:  NR  CCS:  NR | 60.6 (13.3) | 59.8 (14.5) | Echo or SPECT | 12 m |
| Perin 2011 | Medical therapy | BMMNC | 2x10^6^ BMMNC | BM aspiration*, IM (EMM) | Mock injection  but no placebo administered | 20 | 10 | NYHA: 2.3 (0.2)  CCS:  3.0 (0.2) | NYHA:  2.6 (0.3)  CCS:  3.0 (0.3) | 37.0 (10.6) | 39,0 (9.1) | Echo (ǂ) | 6 m |
| Perin 2012a | Medical therapy | BMMNC | 100x10^6^  BMMNC | BM aspiration*, IM (EMM) | Injection of saline with 5% human serum albumin | 54 | 28 | NYHA: NR  CCS:  NR | NYHA:  NR  CCS:  NR | 34.7 (8.8) | 32.2 (8.6) | Echo | 6 m |
| Perin 2012b | Medical therapy | ALDH+  cells | 2.94 (1.58) x10^6^ ALDH+ cells | BM aspiration*, selection of ALDH+ cells by cell sorting  IM (EMM) | Injection of saline with 5% human serum albumin | 10 | 10 | NYHA: 2.5 (0.5)  CCS:  2.0(0.5) | NYHA:  2.6 (0.5)  CCS:  2.5 (0.5) | 36.1 (10.9) | 32.1 (10.6) | Echo (ǂ) | 6 m |
| Pokushalov 2010 | Medical therapy | BMMNC | 41 (16) x 10^6^ BMMNC | BM aspiration*, IM (EMM) | No additional therapy | 53 at 6 months, 49 at 12 months | 46 at 6 months, 33 at 12 months | NYHA: 3.3 (0.2)  CCS:  3.1 (0.4) | NYHA:  3.5 (0.1)  CCS: 3.5 (0.5) | 27.8 (3.4) | 26.8 (3.8) | Echo | 12 m |
| Tse 2007 | Medical therapy | BMMNC | 10-20 x 10^6^ BMMNC | BM aspiration*, IM (EMM) | Injection of saline with 10% human serum albumin | 19 | 9 | NYHA: 2.8 (0.8)  CCS:  3.3 (0.5) | NYHA:  2.8 (0.4)  CCS: 3.1 (0.3) | 51.9 (8.5) | 45.3 (8.3) | MRI | 6 m |
| van Ramshort 2009 | Medical therapy | BMMNC | 98 (6) x 10^6^ BMMNC | BM aspiration*, IM (EMM) | Injection of saline with 0. 5% human serum albumin | 24 | 25 | NYHA: NR  CCS:  3.0 (0.6) | NYHA:  NR  CCS: 2.9 (0.7) | 56.0  (12.0) | 54.0  (10.0) | MRI | 6 m |
| Wang 2010 | Medical therapy | CD34+ cells | 5.6 (2.3) x 10^7^ CD34+ | BM aspiration* and selection of CD34 by MACS, IC | Injection of saline with human serum albumin | 56 | 56 | NYHA: NR  CSS:  3.3 (24.7) | NYHA:  NR  CSS:  3.5 (26.2) | NR | NR | - | 6 m |

BM = bone marrow, BMMNC = bone marrow mononuclear cell(s), CCS = Canadian Cardiology Society functional classification of angina, Echo = echocardiography, EMM = ElectroMechanical Maping, usually using the commercially available NOGA^TM^ system, G-CSF = granulocyte colony stimulating factor, IC= intracoronary, IM=intramyocardial, LVA = left ventricular angiography, LVEF = left ventricular ejection fraction, MACS= magnetic activated cell sorting; MRI = magnetic resonance imaging, NR = Not reported, NYHA = New York Heart Association functional classification of heart failure, SC = stem cells, SD = standard deviation, SPECT = single photon emission computed tomography. (*) BM aspiration = bone marrow was harvested by aspiration and mononuclear cells were isolated by Ficoll density gradient centrifugation. (†) in the meta-analysis; controls from the Losordo 2011 trial were divided into two equal groups to enable separate analysis of each of the BMSC treatment groups. (ǂ)LVEF was measured by ECHO, SPECT and LVA or ECHO and LVA. Results from ECHO are reported here.
